# How Good is the Science That Informs Government Policy? A Lesson From the U.K.’s Response to 2020 CoV-2 Outbreak

**DOI:** 10.1007/s11673-021-10130-2

**Published:** 2021-10-14

**Authors:** Jessica Cooper, Neofytos Dimitriou, Ognjen Arandjelovíc

**Affiliations:** University of St Andrews North Haugh, KY16 9SX Fife, St Andrews, Scotland UK

**Keywords:** COVID-19, epidemic, Methodology, Modelling, Decision-making, Public policy

## Abstract

In an era when public faith in politicians is dwindling, yet trust in scientists remains relatively high, governments are increasingly emphasizing the role of science based policy-making in response to challenges such as climate change and global pandemics. In this paper we question the quality of some scientific advice given to governments and the robustness and transparency of the entire framework which envelopes such advice, all of which raise serious ethical concerns. In particular we focus on the so-called Imperial Model which heavily influenced the government of the United Kingdom in devising its response to the COVID-19 crisis. We focus on and highlight several fundamental methodological flaws of the model, raise concerns as to the robustness of the system which permitted these to remain unchallenged, and discuss the relevant ethical consequences.

## Introduction

The coronavirus disease 2019 (COVID-19) epidemic, caused by severe acute respiratory syndrome coronavirus 2 (SARS-CoV-2) has placed the scientific community in the spotlight. The repeated claims and emphasis of governments across the world on science-based decision-making raise important political and ethical questions. Nevertheless, before these can be addressed with credibility and implemented in practice, it is imperative that the assumptions they rest on, and which regard the relevant material objective issues, are derived through good science (Goldacre 2010), that is scientific inquiry which is methodologically sound and rigorous (Smaldino and McElreath 2016). If scientists make claims which are used to drive or justify drastic social measures that alter the usual course of life and those claims turn out to be vastly inaccurate, the generally high level of trust in scientists (MORI 2017) and their credibility can be fatally undermined, effecting damage to all: research, the scientific community, and the beneficiaries of scientific advice, such as the general public. Thus, in the present article we examine the quality of the specific modelling work by Ferguson et al. (2020) (henceforth referred to as the Imperial Model) which was vastly influential in directing the United Kingdom’s policy decisions in response to the COVID-19 crisis.

Right at the outset it is important to clearly delineate the scope, premises, and limits of our analysis. Firstly, none of the criticisms raised herein emerge from post hoc acquired knowledge, that is we only discuss the quality of science given the information available at the time, rather than retrospectively, i.e. in hindsight. Indeed, most of the criticisms we level relate to the fundamental, methodological aspects of the work. Secondly, our analysis should not be interpreted as criticism of any specific policy. It is imperative to understand that policy-making inherently involves the consideration of extra-scientific issues, for example of axiological and moral nature, to say nothing of the fact that the relevant scientific considerations involve a much broader range of disciplines than epidemiology, such as economics, sociology, behavioural science, and many others.

## Major Methodological Criticisms

The aim of the present article is not to analyse exhaustively all aspects of the Imperial Model. Rather we focus on a few keystone modelling choices and explain the fundamental methodological concerns associated with them. In this section these are examined one by one in turn.

### The Trouble With R

Throughout the ongoing crisis much emphasis, both in the context of policymaking and the understanding of the relevant epidemiology, has been placed on the so-called R number, i.e. the effective reproduction number of the infection (not to be confused with R0, the basic reproduction number which itself is a source of much confusion even in the scientific community [Delamater et al. 2019]). The reason for this is quite clear: the effective reproduction number is the number of new cases of infection in the population generated from its current state. In this section we would like to highlight two main (but by no means the only two) methodological flaws in the modelling of Ferguson et al. (Ferguson et al. 2020) which pertain to the R number: (i) the failure to account for the so-called friendship paradox, and (ii) the inherent bias in the manner the R value is estimated.

#### The Friendship Paradox

The friendship paradox is a well-known phenomenon first reported by Feld (1991) in social networks. Succinctly, the apparent paradox stems from the observation that on average most people have fewer friends than their friends do. It is readily explained by the observation that popular individuals (those with many friends) are more likely to be in one’s friends group than those who are unpopular. The same phenomenon applies in many other networks defined by relationships and unsurprisingly invited a lot of study in the spread of disease in particular (Amaku et al. 2015; Christakis and Fowler 2010). Despite the major effects that the phenomenon can have across relationship networks of different structures irrespective of the R number, it is virtually unaccounted for by the Imperial Model. The only, vastly over-simplistic and coarse due paid to it, comes in the form of assumptions (we discuss how different assumptions are justified and approached more generally in Section 2.2) on sub-population interaction frequencies in a small number of different environments (e.g. schools or workplaces) (Ferguson et al. 2020).

#### Inherent Bias in the R Number

The next key omission in the treatment of the reproduction number is a subtler one and it concerns the discord between the manner in which the number is estimated and the manner in which the said estimate is thereafter employed in prediction. To start with the former, the R number estimate is based on population level statistics of, amongst others, recorded deaths, hospital admissions, and positive tests for the virus, over time. Thus, to state the obvious, the estimate is based on the dynamics of the disease amongst those who have already been infected. The important subtlety to observe here is that this is invariably not a randomly drawn subset of the population. Rather, if certain sub-populations are more prone to infection, it is the effective reproduction rate amongst this sub-population that is being estimated, which is always higher than what the effective reproduction rate would be across the entire population. This observation has important consequences on modelling predictions. Firstly, it is clearly fallacious to assume the same R number to arbitrary non-infected individuals–the predictions of the numbers of deaths, hospitalizations, etc. are all going to be biased upwards, i.e. be overestimates. Moreover, the actual statistics for any of the aforementioned are bound by the size of the susceptible demographic. In the absence of the relevant virological understanding of this size, or more precisely the distribution of susceptibility (in a broad sense) across the population, the predictions of a model which ignores this fact cannot be taken seriously. Yet, this is precisely what we saw with the work of Ferguson et al.

To give a specific example as a means of illustrating our point, consider the scenario whereby lockdown is introduced, of the type indeed adopted by the government of the United Kingdom. What can be expected to happen is a drastic reduction in the transmission of the virus in the general population and with it a dramatic drop in the R number (which is indeed what has been observed). Following this initial period, it is possible that significant transmission is confined to certain environments only, such as hospitals and care homes for the elderly. Since, as noted earlier, the R number is estimated from those already infected, this means that the number can rise even to the levels preceding the lockdown. However, as already explained, the infection now by design, so to speak, being confined to a specific (and small in size) sub-population, this infection rate has little bearing on the risk for the general public as a whole.

As emphasized right at the outset of the present article, our analysis focuses on the quality of the modelling decisions in the context of the information available at the time, i.e. without the benefit of hindsight, and the lack of accounting for the highlighted phenomenon is a major flaw regardless of the actual unfolding of events thereafter. Nevertheless, it is interesting to observe that the aforementioned bias may be used to explain what to some is the surprisingly good outcome of the Swedish policy. Recall that Sweden did not impose a lockdown but merely voluntary social distancing recommendations, keeping restaurants, cafes, bars, and many schools open. Yet, as of the end of May (when the United Kingdom started relaxing the lockdown) Sweden had recorded 4,350 deaths with COVID-19, compared to at least 39,045 in England—circa 4.2 vs 7.0 deaths per 10,000 people. In short, it is likely that the subset of the population at high risk is relatively small and the initially exponential rise in infections and deaths inherently peaks far earlier than in a homogeneously susceptible population.

### Assumptions, Latent Variables, and Absence of Validation

As with virtually any practical epidemiological model, the model used by Ferguson et al. contains a number of latent parameters. These parameters describe (explicitly or implicitly) various characteristics of the disease itself, as well as relevant characteristics of the population studied, interaction patterns within it, etc., but which are not directly observable (unlike, say, the number of excess deaths). Rather than being directly observed, latent parameters have to be either (i) “manually” pre-set based on the best understanding of the relevant phenomenology (with a further caveat which will be discussed shortly), or (ii) inferred from observable data (e.g. through the usual process of validation on withheld data and maximum likelihood hypothesis choice). Alarmingly, neither of these two approaches were followed in the setting of the parameters in the model of Ferguson et al.

As just suggested, the list of latent parameters which were set in a rather ad hoc manner by Ferguson et al. is rather long (e.g. the value of R0, relative infectiousness of symptomatic vs asymptomatic individuals, the parameters and type of distribution of individuals’ infectiousness, etc.) and there is little benefit to discussing them all exhaustively given that our aim is to highlight the general methodological concerns regarding the work. Instead, we highlight an illustrative example, and having already introduced it previously, we may as well use for this purpose the R number (noting that the issues discussed now are different from those which were at the focus in the previous section). In particular, what the authors of the original report did was to adopt the uniform R value of 2.4, which is the estimate based on early epidemiological modelling (Riou and Althaus 2020) applied on the first 425 recorded cases in Wuhan (Li et al. 2020). Considering that the effective reproduction number is a function not only of the relevant virological factors (e.g. how infectious the virus is) but also of environmental and social variables (amongst others: weather (Tan et al. 2005), population demographics, number and nature of social interactions, and individuals’ hygiene practices), in which Wuhan and the United Kingdom differ significantly, this is clearly an entirely inappropriate modelling choice. Given when the report was published, it is also a most curious choice too: there had already been sufficient data from the United Kingdom which could have been used or indeed data from a number of other European countries which would have been more credibly argued as a sound starting point (Saglietto et al. 2020; Roques et al. 2020).

### System Complexity and Sensitivity Analysis

In no small part, the challenge of modelling the spread of infectious disease emerges from the complexity of the modelled system. As we noted in the previous section, the evolution of the system is governed by a large set of parameters including population demographics, social interaction patterns, infection susceptibility variability, and many others, with intricate interactions between these and highly non-linear effects on the ultimate outcomes of interest (e.g. the number of hospitalizations or deaths). A consequence of this complexity is that in some cases reliable inference or validation of model parameters is not possible, for example due to the unavailability of sufficient amounts of data required for validation. With respect to this, as a note of elementary principle, it is firstly important to note that such limitations do not give the modeller the licence to make ad hoc choices and then use the emergent predictions as basis for recommendations on, say, policy issues. A model which is known not to be underlain by soundly set parameters is untrustworthy. What can be done, however, in an effort to gain as much insight into the model and the modelled phenomenon as possible, is to subject the predictions to sensitivity analysis (Blower and Dowlatabadi 1994; Drechsler 1998). In other words, the principle is that of examining the relationship between model parameters and the ultimate predictions. If this sensibility with respect to a particular parameter (or indeed a set of parameters) is low, an argument can be made that even if its exact value is not known, the corresponding effect on the prediction is low, leading to a possibly useful model and instructive error bounds. On the other hand, if the aforementioned sensitivity is high, i.e. if small changes to a latent parameter effect large changes to the ultimate predictions, the model can be rejected as one of little practical use. Worryingly, little such analysis was performed by Ferguson et al.—the sensitivity to most parameters was not considered at all while for a couple of others only a highly limited range of values was examined (e.g. R0 between 2.0 and 2.6).

### Oversimplicity of Virological Modelling

As was known from just about the very onset of the disease, COVID-19 is caused by a single-stranded RNA virus (SARS-CoV-2). This type of virus is well-known to be highly susceptible to copying errors (Duffy 2018) and thus the development of new strains, the prevalence of which in the population is driven by environmental factors (including social policy, individuals’ behaviour, weather etc.). New mutations can significantly differ in how infectious or deadly they are in comparison with the known ones (or more broadly, the existing ones), as well as in various other characteristics such as when the peak of infectiousness of an individual occurs relative to the onset of symptoms, asymptomatic transmission, etc.

While there can be little doubt that the modelling of this phenomenon is most challenging indeed, the failure to account for it should be itself sufficient to reject a model for making concrete predictions which affect policy (be it directly or indirectly, e.g. by affecting the mood of the public, the perceived risk, etc.). In a sentence, the possibly insurmountable difficulty of accounting for an important factor in modelling does not give one the licence to omit it while knowing that the predictions may be a very poor match to reality.

The importance of high mutability of single-stranded RNA viruses is particularly important in the context of the kind of policy decisions which were made based on modelling such as the one primarily discussed herein. In particularly, under what one may call “normal” circumstances, the prevalence of strains which have more severe health effects (including death) tends to reduce over time for a simple reason: the ability of very ill people to go about their normal lives is (virtually by definition) reduced and so is (on average) their contact with other individuals. Severely ill people will not partake in social and leisure activities, will limit or displace their shopping, etc. On the other hand, those infected with strains which cause milder illness, or indeed no symptoms at all, will change their behaviour little, if at all, increasing the prevalence of the infection with the strain. However, environmental changes, say such as those affected by the imposition of a lockdown, alter the picture, possibly slowing down the downward spiral of the prevalence of the more severe strains.

## Ethical Implications

In the previous section we described some of the most serious methodological flaws in a crucial report that influenced the U.K. government’s response to COVID-19. The nature and seriousness of these flaws, that is the fact that they went uncorrected, firstly highlights major structural challenges with the manner governments obtain scientific advice, crucial in, if not every then virtually every policy made today. Questions such as whether the body of scientist advisers is representative of the scientific community at large, whether the breadth of their expertise domains is adequate for the specific problems faced by the government, as well as whether the advisers have sufficient autonomy and freedom to voice their genuine opinions, should all be asked and investigated.

Another particularly worrying ethical consequence of the Imperial Model, and thus also of the system which allowed the aforementioned methodological flaws to pass unchallenged, concerns the effect that its predictions had on the overall perception of the pandemic in the country (and wider; the model has been cited as influencing public policies in other countries as well)—the legislature and the public alike. The catastrophic predictions of the model, publicized widely by politicians, the mainstream media, and in most cases genuinely concerned but lay members of the public, have affected public policy decision-making both directly and indirectly. The predictions have served to increase the political cost of harm associated with COVID-19, thus de facto devaluing harm from other causes. Indeed, the profoundly negative impact of COVID-19 measures on the mental health of the population has been widely documented (Pfefferbaum and North 2020; Liu et al. 2020) as has the in- crease in non-CoOVID related mortality and morbidity (Docherty et al. 2020; Maringe et al. 2020).

Further to health-related ethical concerns, are concerns which regard political decision-making and legislation. The backdrop created by the Imperial Model’s predictions has facilitated a narrative which unduly elevates science, that is scientists, to the policy-making role epitomized by the oft-repeated, yet meaningless slogan “following the science” (or a variation thereof) (Mercuri 2020). Science, by its very nature and realm of inquiry, cannot fulfil this duty for policy-making demands value-based judgements, which are inherently extra-scientific (Arandelovíc 2021). This was recently expressed well by Lavazza and Farina (2020):

Given the dangerousness and the extent of the contagion, almost no one has questioned the suggestions that these experts have advised policymakers to implement. Quite often the latter explicitly sought experts’ advice and justified unpopular measures (e.g., restricting people’s freedom of movement) by referring to the epistemic authority attributed to experts. (1)

Echoing our thoughts on the matter, they go on:

… when values are involved it is no longer just a matter of finding the “best technical solution,” but also of making discretionary choices that affect citizens and that cannot be imposed solely on the basis of epistemic authority. (1)

Yet, this elevation of technical authority was not uniform or consistent. Scientists who voiced disagreements over the government’s choices and who proposed alternative means of response to the crisis, such as Kulldorff et al. (2020), have, in spite of their established technical credibility, been side-lined at best, and not infrequently vilified as COVID deniers, as uncaring individuals, as people willing to let the elderly die^1^, etc. Not only is this response logically inconsequent and unfair to these scientists but it is also egregiously dangerous—a lack of freedom to debate scientific questions can only stifle our understanding of a phenomenon and thus impair our ability as a society to deal with the challenge we face. As Mercuri (2020) asks: “Is it fair to say one is ‘following the science’ if only a small group of scientists or disciplines is included in the conversation?” (1576).

The same risk aversion unduly directed towards a single source of risk, namely the disease (COVID-19) itself, which was instrumental in guiding policy decisions in response to the epidemic, has also profoundly affected the policymaking process itself. Mirroring the aforementioned phenomenon in this aspect as well, the highly troubling manner in which this has happened passed with little notice by most. Lord Sumption, a highly distinguished intellectual and a former senior judge who sat on the Supreme Court of the United Kingdom between 2012 and 2018, was one of a small number of prominent individuals who spoke out. In addition to highlighting the importance of non-scientific factors in public policy decision-making that we discussed earlier, Lord Sumption described a number of legal transgressions of the state (both its legislative and executive elements), including a lack of legal basis for a number of rules imposed on the population Sumption (2020). In most circumstances, actions like these would have provoked a vociferous public outcry. That they did not do so in this case, at least initially, does not reflect an indifference of the public—quite on the contrary. The initial reaction of the British public, that of quiet obedience (Jackson et al. 2020), was made possible only by presenting as the alternative choice the path to the cataclysmic state predicted by Ferguson et al. As noted by Furedi (2008):

… the current conceptualization of resilience assumes that vulnerability is the defining condition of social life. One likely consequence of this approach is the reinforcement of the passive side of public life. (645)

Unsurprisingly, when in time the public perceived a discrepancy between what “the scientists” (in inverted commas, to highlight the simplistic and misleading messaging of the government and the media, as discussed earlier) were predicting and the actual state of affairs, the behaviour of the public changed. Indeed, Smith et al. (2020) found: “Decreased perceived effectiveness of lockdown measures was linked to non-adherence” and “Non-adherence was also linked with decreased perceived severity of COVID-19” (41).

At the start of this article, we reflected the data which consistently over a period of years show that the scientific community enjoys high regard from the general public (see Section 1). However, this trust should not be taken for granted. It is not difficult to see how many of the actions of scientists which we raised concerns about in this section can only serve to erode the said trust (Arandelovíc 2017). Some of these actions may be genuine mistakes. While these mistakes may point to structural problems, which ought to be addressed in due time, the mistakes themselves should be judged with caution, balance, and humility—mistakes do happen. However, dishonesty about mistakes should not be accepted and it is actions of this nature that can effect the greatest harm by virtue of long-term loss of confidence in scientists or worse yet, science itself. The manner in which mortality has been reported during the crisis provides a poignant example. Initially, amongst other data, the COVID-19 Data Dashboard (see https://coronavirus.data.gov.uk/) reported for England all deaths of people who have ever had a positive CoV-2 test; subsequently a change was made to include only those individuals who have had a positive within 28 days of death (the reasons for the change are largely inconsequential here, though the reader will probably be able to infer them following the reading of the present paragraph). This (the former more so than the latter) is a perfectly principled way of recording mortality for the purposes of surveillance or the analysis of COVID-19 specific mortality. However, it is entirely incorrect to describe these deaths as being “from COVID-19.” Only a robust statistical analysis can provide a sound estimate of the proportion of the deaths recorded in the manner described which are attributable to the disease. Yet, we found that in the period of up to September 2020, out of 14,260 references to COVID-19 mortality on the BBC web site, 10,050 (> 70 per cent) used the phrasing “died from” or “died of” rather than “died with.” We found the same on all mainstream media websites; to give another example, out of 1637 references in the same period on *The Telegraph* web site, 1415 (> 86 per cent) said “died from” or “died of.” While this could at first sight be brushed off as a reflection of journalists’ lack of scientific and statistical training, even a slightly more careful examination of the issue reveals a different picture—the same justification cannot be employed to defend some highly prominent and accomplished scientists who repeatedly use the same phrasing. Consider the words of Prof. Spiegelhalter on BBC’s Newsnight (18 December 2020):

I was surprised to see that more people have been killed by COVID-19 than civilians were killed in the U.K. during the Second World War. That is 64, 000 were killed in 6 years. We have had more than that who have died from COVID. (pp, all emphasis is ours)

Spiegelhalter was not referring here to any sophisticated statistical analyses or estimates but rather the raw “died with COVID” figures at the time. Such distortion of truth cannot be a mistake, not coming from somebody as scientifically credible as Spiegelhalter. Thus, and relating this to our aforementioned discussion of changes in the public’s obedience of the government’s COVID-19 measures, it is difficult to escape the conclusion that this is a purposeful attempt to make observations fit predictions.

## Conclusions

The overarching aim of this article was to bring to attention a number of serious concerns about the quality of scientific advice used by governments in making important policy decisions and highlight the serious ethical consequences. Our specific focus was on the recent modelling work done by Ferguson et al. at Imperial College London, which was instrumental in driving the U.K. government’s handling of the ongoing COVID-19 epidemic. In particular, we discussed a number of major flaws in the aforementioned modelling. Importantly, none of our criticisms rely on post hoc knowledge, i.e. on knowledge unavailable to the modellers at the time. Rather they all concern issues which are of a fundamental, methodological nature. Some of these are subtler than others, but all of them affect the model’s predictions in a manner which render its practical usefulness all but void.

Our hope is that the issues we raised will be taken constructively and in a manner which will effect improvement in future. Most proximally, processes which ensure better quality scientific advice should be put into place, as policymaking premised on ill-founded science cannot itself be good. Specifically, the presented analysis of a number of serious methodological flaws highlights the need for more openness and scrutiny of scientific advice given to governments, for example by instituting something akin to peer review. Secondly, given that policy decisions influenced by the Imperial Model have already been made and that the effects of these decisions continue to be assessed in ways which are underlain by at least some of the flawed assumptions and modelling choices we highlighted, particular care has to be taken in interpreting observations as the undertaken measures are phased out.

## Footnotes

1 See e.g. https://www.investorschronicle.co.uk/news/2020/12/18/how-covid-19-could-lead-the-way-to-better-healthcare/.
